# Evidence for the early Toarcian Carbon Isotope Excursion (T-CIE) from the shallow marine siliciclastic red beds of Arabia

**DOI:** 10.1038/s41598-022-21716-0

**Published:** 2022-10-27

**Authors:** Mahmoud Alnazghah, Ardiansyah Koeshidayatullah, Abdulkarim Al-Hussaini, Abduljamiu Amao, Haijun Song, Khalid Al-Ramadan

**Affiliations:** 1 Exploration Organization, Saudi Aramco, Dhahran, Saudi Arabia; 2 Department of Geosciences, College of Petroleum Engineering and Geosciences, King Fahd University of Petroleum and Minerals, Dhahran, Saudi Arabia; 3 Center for Integrative Petroleum Research, College of Petroleum Engineering and Geosciences, King Fahd University of Petroleum and Minerals, Dhahran, Saudi Arabia; 4 State Key Laboratory of Biogeology and Environmental Geology, China University of Geosciences, Wuhan, China

**Keywords:** Geology, Stratigraphy, Sedimentology, Solid Earth sciences, Geochemistry

## Abstract

The Toarcian Oceanic Anoxic Event (T-OAE) and its corresponding Carbon Isotope Excursion (CIE) have been reported widely across the Tethyan region and globally. In Arabia, and based on ammonite dating, the time window of the T-OAE coincided with the deposition of the reddish siliciclastic unit of the Marrat Formation. However, no evidence of the T-OAE/CIE was ever reported from Arabia because these red beds were previously interpreted as continental deposits. Recently, these red beds have been recognized as shallow marine deposits which opened an opportunity to assess the occurrence and expression of T-OAE–CIE in Arabia. In this study, a multiproxy geochemical characterization was performed on the Toarcian Marrat Formation to infer the chemistry of the paleowater column and identify intervals of possible T-OAE/CIE in Arabia. While the low concentrations of redox-sensitive elements (Mo, U, V, Cr) may indicate a shallow oxic marine settings, the coupled negative δ^13^C_organic_ excursion and apparent increase in the chemical weathering suggests that the deposition of Marrat red beds coincided with the development of T-CIE and possibly time-equivalent to the T-OAE globally. The origin of reddening is interpreted to have occurred during the middle Marrat deposition due to the stabilization of unstable hydrous iron oxides to hematite under oxic marine conditions. The proposed model further indicates the possible development of source rocks in the deep, anoxic environment counterpart where the T-OAE may be expressed. Since our study documents the first record of the T-CIE and discuss the origin of shallow marine siliciclastic red beds in the Arabian Plate, this will have significant implications for the overall understanding of the T-CIE globally and for hydrocarbon exploration through realizations of potential new source rocks associated with the OAEs in the Toarcian and other time intervals.

## Introduction

The Toarcian Oceanic Anoxic Event (T-OAE) is one of the key biogchemical events across the geological record. Previous studies have argued that the T-OAE was triggered by the emplacement of the Karoo Large Igneous Province coupled with the release of carbon from climate-sensitive reservoirs (e.g., gas hydrates, wet lands, permafrost soil), causing severe environmental perturbations^1–7^. Environmental perturbations (such as carbon cycle perturbation, increasing *p*CO_2_, and global warming) caused major faunal extinctions during this time interval^3,7–16^. While the global extent of the T-OAE was questioned because most datasets were documented from the epicontinental seas of northwestern Europe^5,9–11^, other studies highlighted the occurrences of time-equivalent negative Carbon Isotope Excursion (CIE) across the Tethys and Panthalassa oceans, advocating the global nature of the T-OAE^17–19^. Globally, the T-OAE is marked by a major negative carbon excursion of δ^13^C_organic_ and δ^13^C_carbonate_^3^, within carbonate strata^20^, and fossil wood^4^. Most of the previous research on the T-OAE focused on deep marine black shales and their time-equivalent shallow-water carbonate facies^2,3,11^. However, recent studies show a consistent occurrence of deep and shallow marine red beds during and after the onset of marine anoxia across the geologic record, significantly increasing the importance of these red beds as possible indicators of OAE’s^21–26^.

**Figure 1:**
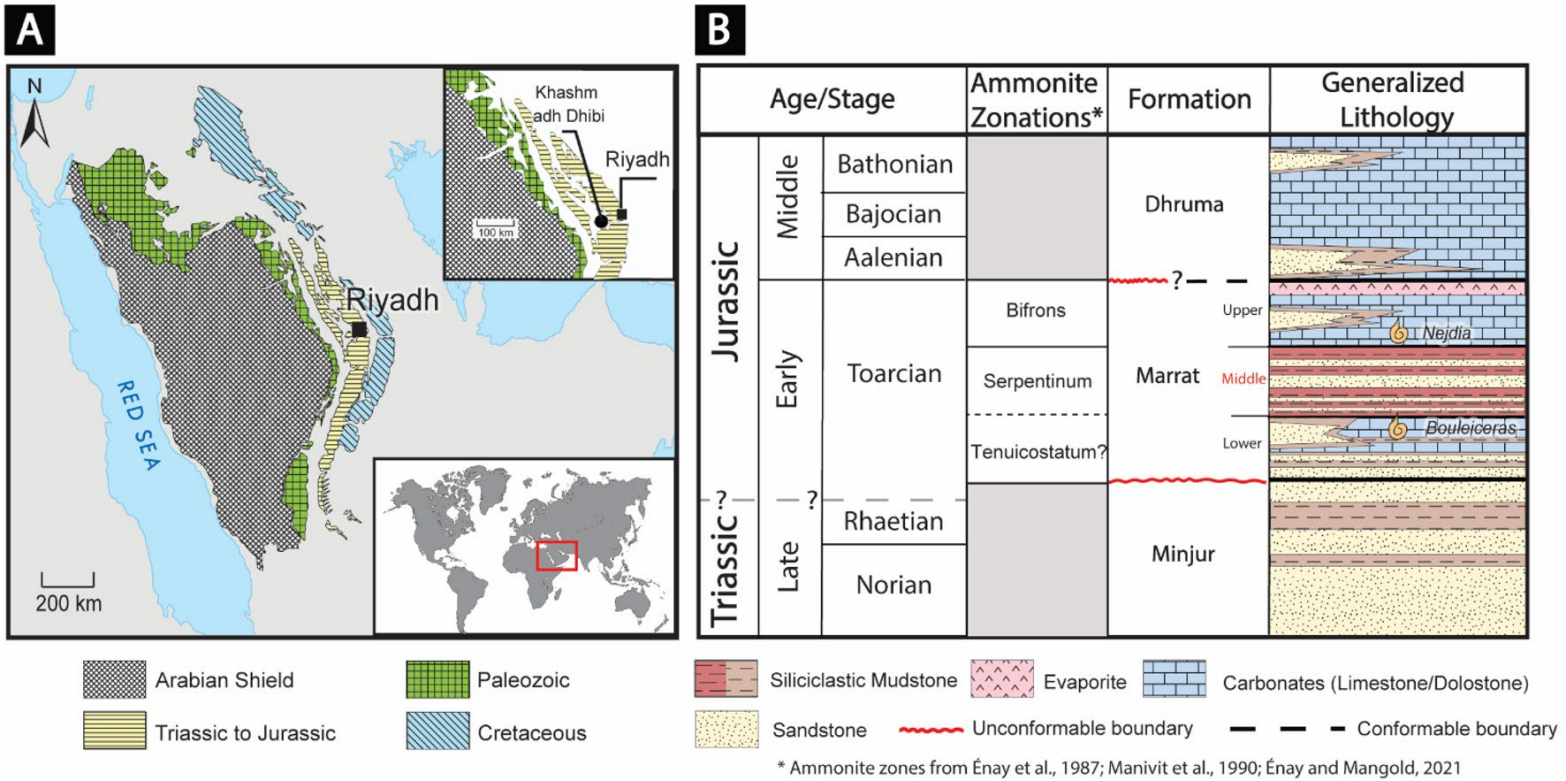
(**A**) Geological map showing the location of the Khashm adh Dhibi outcrop where the stratigraphic section was measured and sampled^35^. (**B**) Generalized stratigraphic column of the Late Triassic—Early Jurassic in Saudi Arabia.

Although the ammonite dating of the Marrat carbonates of Arabia constrains the age of the middle Marrat siliciclastic red beds to be within the time window of the T-OAE (Fig. 1)^27–29^, no documentation or attempts have been made to investigate whether this globally recognized event had extended to Arabia. This can possibly be explained by the fact that these red beds have long been viewed as continental deposits^30–34^ with the assumption that they cannot yield important geochemical results that can be used for correlation with the reported T-OAE/CIE data from the nearby Tethyan basins. Recently, however, a revised interpretation of these red beds indicated that these red beds were deposited under shallow-marine settings^35^, providing a unique opportunity to assess, for the first time, the possible extent of the T-OAE/CIE across the Arabian Plate and the possibility of using shallow marine siliciclastic red beds as an indicator of OAEs or CIE. Therefore, this study aims to (i) assess whether the T-OAE/CIE event influenced the Arabian Plate and (ii) reveal whether there is any record that can be attributed to environmental changes associated with the T-OAE/CIE through systematically conducting high-resolution multiproxy geochemical characterizations of the Marrat Formation. The outcomes of this study are expected to help enhance the global understanding of the T-OAE/CIE and how this event is represented in shallow-marine siliciclastic deposits.

## Geological settings and study area

During the late Permian to early Triassic, the Zagros rifting had a profound impact on the Arabian Plate and the sedimentary architecture of its Mesozoic strata^36,37^. The opening of the Neo-Tethys ocean, as a result of the Zagros rifting, had resulted in the development of an ENE-dipping passive margin along the northeastern margin of the Arabian Plate^36–38^. In addition to the Zagros-related ENE passive margin, another N-dipping Neo-Tethys passive margin was formed along the northern edges of the Arabian Plate due to the Early Jurassic back-arc rifting in the eastern Mediterranean^36–38^. The Early Jurassic Marrat Formation is forming a discontinuous, N-S oriented, arc-shaped outcrop belt that can be traced for more than 650 km in central Arabia, with a total thickness that ranges between 111 and 142 m^30,31^. It has an unconformable contact with the underlying Triassic Minjur Formation, while the upper contact with the overlying Dhruma Formation is generally found to be conformable^30,31,39–41^. However, some published articles suggest that the Marrat-Dhruma contact is unconformable in some places^30,31,34,42,43^. Previously conducted studies on the Marrat Formation divided its deposits into three lithological units; lower, middle and upper^30,31,39,40,42,44–46^. The lower Marrat consists of siliciclastic deposits at the base that transition vertically into carbonates, while the upper Marrat unit is mainly composed of carbonates and anhydrites^30,31,35,39,40,42,44,45^. The middle Marrat is consistently made of reddish mudstones (claystone) with intercalations of sandstone and siltstone^30,31,35,39,40,42,44–46^. The lower and upper Marrat carbonates are replaced by siliciclastics deposits towards the southern parts of the outcrop (towards the updip direction)^30,31^.

The ammonite dating of the Marrat carbonates constrains the age of the middle Marrat red beds to be within the *serpentinum* ammonite zone of the early Toarcian^27–29,47^. The lower Marrat carbonates, where the *Bouleiceras* and *Protogrammoceras* faunas were found, are assigned to be representing the upper part of the *Tenuicostatum* zone to the lower part of the *Levisoni* (or *Serpentinum*) zone of the Mediterranean and northwest Europian scales^27,29^. For the upper Marrat carbonates, the identified *Nejdia* fauna is suggessting middle Toarcain age (*Sublevisoni* Subzone of the *Bifrons* Zone)^27,29^.

Even though the outcrops of the Marrat Formation are exposed at different localities, the Khashm adh Dhibi locality is considered to be the reference section due to the well-preserved exposures that it hosts^30,31,39^. Furthermore, the existence of detailed biostratigraphic (ammonite) analyses on the Khashm adh Dhibi section gives it a competitive advantage over the other Marrat sections^27,28,47^. Therefore, and based on the aforementioned objectives of our study, the Khashm adh Dhibi section (24°19′ 54′′ N; 46°06′ 38′′ E) was selected to be analyzed in this study.

## Methods

**Figure 2:**
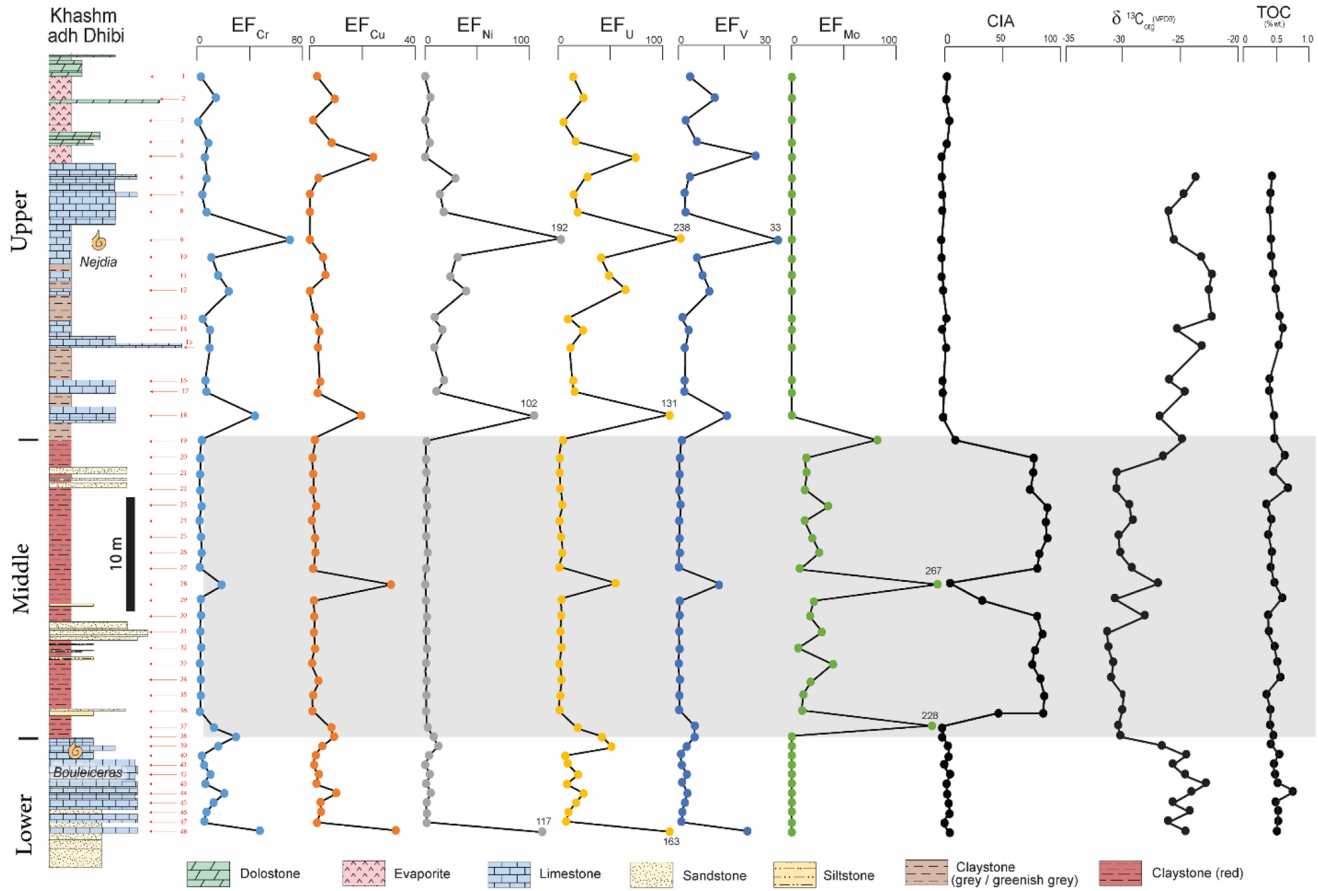
The measured section of the Marrat Formation in Khashm adh Dhibi with sample locations (red arrows) with plots of the calculated enrichment factors (relative to average shale) of some redox sensitive elements (Cr, Cu, Ni, U, V, Mo), Chemical Index of Alteration (CIA), δ^13^C_org_, and TOC. It is notable to highlight that the lowermost siliciclastic parts of the lower Marrat is not included in this measured section.

A stratigraphic section of the Marrat Formation was measured at Khashm adh Dhibi, central Arabia (Figs. 1 and 2) and was sampled at approximately 1-m intervals for high-resolution elemental, mineral, Total Organic Carbon (TOC), and stable organic carbon isotope analyses. Prior to the Inductively Coupled Plasma (ICP) analysis, the collected samples were grounded to very fine powder (to less than 10 micron) using an agate grinder to avoid sample contamination, and then they were liquefied using the alkali fusion preparation technique. Here, 6 M hydrochloric acid (HCl) was used to dissolve the samples. In addition, 10 ml of de-ionized water were added into the solution to ensure that total dissolved solids will be less than 0.1%. The solutions were then further acidified by 2% HNO_3_ and filtered to remove particles > 0.45 μm^48,49^. Elemental concentrations were measured using Agilent 7500ce Inductively Coupled Plasma Mass Spectrometry (ICP-MS), after using standard single- and multi-element references materials for calibration. Detection limits for this analysis is as low as 0.01 ppb in solution under the usual operating conditions. Mineralogical identification using X-ray Diffraction (XRD) was conducted using an InXitu BTX 308 XRD Analyzer. After isolating the organic matter from the collected samples through acid (HCl-HF) maceration, TOC and δ^13^C_org_ (δ ^13^C of the organic matter) analyses were performed using a Costech 4010 Elemental Analyzer combustion system coupled with Thermo DeltaV Plus Isotopic Ratio Mass Spectrometer. The δ^13^C_org_ values are reported in the Vienna Pee Dee Belemnite (VPDB) standard and have analytical precision of 0.1‰.

## Results

### Mineralogy

The Marrat red beds are composed primarily of phyllosilicate (up to 59%) and quartz (up to 99%) with an average of 17% and 32%, respectively, in addition to minor amounts of calcite and dolomite (< 5%). Additionally, notable increases in the contents of pyrite (up to 4.4%) and hematite (up to 8.1%) were observed in the red beds. In contrast, the bounding carbonates are predominantly composed of calcite, constituting up to 99.3% of the rock volume, with minor amounts of quartz (< 3%), pyrite and hematite (< 1%).

### Redox sensitive elements

Concentrations of some redox-sensitive elements (e.g., Cr, U, V, and Mo) were analyzed and normalized to aluminum across the Marrat Formation (Table 1 and supplementary material). In addition, enrichment factors (EF)^50^ (to average shale^51^) were calculated for some of the redox-sensitive elements (Fig. 2) (detailed calculation and equation are available in the supplementary material). In general, similar trends are observed in the normalized redox-sensitive elements (Cr, U, and V) concentrations across the lower, middle, and upper units of the Marrat Formation where these elements are enriched in the upper and lower Marrat carbonates and no enrichment across the middle Marrat red beds (Table 1 and supplementary material). However, Molybdenum (Mo) is an exception where it shows an inverse signal to the aforementioned elements (Table 1). Across the middle Marrat red beds, the average normalized Cr, U, and V concentrations are 4.7, 0.07, and 11.9, respectively (Table 1). In contrast, the upper and lower carbonates show a significant enrichment (up to 7x) of these elements with average values of 15.9 and 16.9 for Cr, 0.37 and 0.19 for U, and 73.7 and 54.9 for V (Table 1).

**Table 1:** Geochemical analyses of the Marrat Formation.

Redox-sensitive elements (after AI normalization)							Enrichment Factor (to average shale)							Chemical and Weathering indices			Stable ^13^C_org_ and TOC	
Ba/AI	Cr/AI	Cu/AI	Mo/AI	Ni/AI	U/AI	V/AI	EF (Ba)	EF (Cr)	EF (Cu)	EF (Mo)	EF (Ni)	EF (U)	EF (V)	CIA	PIA	CIW	^13^C_org_	TOC
																	VPDB	% wt
**Upper Marrat carbonate**																		
0.00	3.30	1.53	0.00	0.00	0.18	25.52	0.00	2.93	2.72	0.00	0.00	15.70	3.99	1.11	1.11	1.11		
27.31	16.12	5.32	0.00	4.22	0.56	41.49	3.77	14.33	9.45	0.00	4.96	25.53	12.14	0.82	0.82	0.82		
11.92	1.03	0.69	0.00	0.00	0.12	10.53	1.64	0.92	1.22	0.00	0.00	6.48	2.56	2.26	1.30	2.28		
5.67	9.70	4.64	0.00	3.71	0.29	29.44	0.78	8.62	8.25	0.00	4.37	18.12	6.25	1.00	0.65	1.00		
0.00	6.75	13.54	0.00	0.00	1.18	122.67	0.00	6.00	24.08	0.00	0.00	75.49	25.60	0.18	0.10	0.18		
91.89	8.28	1.83	0.00	24.59	0.18	47.82	12.67	7.36	3.26	0.00	28.93	29.43	3.92	0.29	0.23	0.29	−23.84	0.46
23.42	4.59	0.00	0.00	11.88	0.10	26.36	3.23	4.08	0.00	0.00	13.98	16.22	2.18	0.53	0.45	0.53	−24.91	0.44
47.24	8.27	0.00	0.00	15.04	0.12	32.52	6.52	7.35	0.00	0.00	17.69	20.01	2.52	0.48	0.35	0.48	−26.24	0.42
158.32	79.42	0.00	0.00	163.47	1.53	387.80	21.84	70.59	0.00	0.00	192.32	238.65	33.12	0.04	0.00	0.04	−25.76	0.44
0.00	12.26	2.80	0.00	26.46	0.29	69.08	0.00	10.89	4.99	0.00	31.13	42.51	6.28	0.21	0.02	0.21	−23.34	0.45
0.00	18.08	3.32	0.00	20.47	0.38	81.22	0.00	16.07	5.90	0.00	24.09	49.98	8.11	0.20	0.13	0.20	−22.51	0.50
32.13	27.16	0.00	0.00	33.23	0.48	106.86	4.43	24.14	0.00	0.00	39.09	65.76	10.34	0.14	0.29	0.14	−22.78	0.54
15.48	4.89	1.01	0.00	7.64	0.07	17.39	2.14	4.35	1.80	0.00	8.98	10.70	1.50	0.87	0.60	0.87	−22.53	0.60
59.00	11.14	2.01	0.00	13.96	0.16	40.76	8.14	9.90	3.57	0.00	16.43	25.08	3.54	0.46	0.43	0.46	−25.61	0.66
22.67	10.71	1.73	0.00	7.35	0.10	21.26	3.13	9.52	3.07	0.00	8.65	13.08	2.23	0.77	0.30	0.77	−23.41	0.59
25.83	7.22	2.23	0.00	15.33	0.10	25.53	3.56	6.42	3.96	0.00	18.03	15.71	2.17	0.63	0.44	0.63	−26.46	0.43
121.87	8.04	1.66	0.00	9.09	0.10	27.72	16.81	7.15	2.95	0.00	10.69	17.06	2.09	0.70	0.36	0.71	−24.92	0.43
233.68	49.51	10.95	0.00	87.54	0.75	213.11	32.23	44.01	19.47	0.00	102.99	131.14	16.13	0.08	0.04	0.08	−27.16	0.51
**Middle Marrat red beds**																		
15.05	4.20	1.06	2.67	1.15	0.06	9.67	2.08	3.74	1.89	82.12	1.35	5.95	1.32	5.07	3.70	5.15	−25.16	0.51
3.36	2.77	0.57	0.45	0.75	0.03	4.79	0.46	2.46	1.02	13.90	0.88	2.95	0.68	69.92	94.29	96.46	−26.85	0.69
2.65	2.67	0.69	0.46	0.92	0.03	4.71	0.37	2.37	1.23	14.13	1.08	2.90	0.55	65.10	72.46	77.86	−30.38	0.44
1.70	2.75	0.61	0.41	0.90	0.02	4.85	0.23	2.45	1.08	12.49	1.05	2.99	0.44	61.35	66.28	72.29	−30.40	0.69
4.90	4.03	1.34	1.13	1.45	0.04	8.36	0.68	3.58	2.38	34.85	1.71	5.14	0.84	77.79	82.22	83.53	−29.27	0.32
2.58	2.19	0.43	0.40	0.77	0.02	4.21	0.36	1.95	0.76	12.34	0.90	2.59	0.42	79.80	95.16	96.15	−28.96	0.41
2.83	3.44	1.09	0.63	0.80	0.02	6.62	0.39	3.06	1.93	19.51	0.94	4.07	0.53	79.43	88.26	89.80	−30.22	0.35
1.62	4.03	1.19	0.85	2.01	0.03	8.50	0.22	3.58	2.11	26.21	2.37	5.23	0.63	71.89	80.82	84.07	−30.08	0.41
1.15	2.40	0.64	0.24	0.66	0.01	3.96	0.16	2.14	1.14	7.52	0.78	2.44	0.30	69.17	77.79	81.87	−29.07	0.39
298.49	21.05	17.32	8.67	0.00	0.63	91.37	41.17	18.71	30.78	266.70	0.00	56.23	13.53	2.66	0.01	2.73	−26.73	0.46
15.26	3.28	0.86	2.64	0.59	0.03	7.44	2.10	2.91	1.52	81.29	0.69	4.58	0.62	19.90	18.17	20.45	−30.56	0.59
3.40	3.48	0.92	0.69	1.39	0.03	6.69	0.47	3.10	1.64	21.23	1.63	4.12	0.57	69.94	79.85	83.87	−27.90	0.35
1.91	2.96	0.84	0.57	1.08	0.02	5.61	0.26	2.63	1.50	17.59	1.28	3.45	0.43	74.48	83.31	85.86	−31.17	0.35
5.70	3.71	1.10	0.93	1.41	0.02	7.32	0.79	3.29	1.96	28.66	1.66	4.50	0.53	66.91	74.09	78.63	−31.04	0.45
0.93	2.42	0.48	0.20	0.68	0.01	3.62	0.13	2.15	0.85	6.13	0.80	2.23	0.29	62.46	66.04	70.31	−30.63	0.50
2.25	3.30	1.82	1.29	1.11	0.03	7.29	0.31	2.93	3.23	39.65	1.31	4.49	0.64	70.31	74.63	77.06	−30.82	0.55
1.92	3.22	0.70	0.58	0.98	0.02	5.59	0.27	2.86	1.25	18.00	1.15	3.44	0.48	77.05	86.59	88.60	−29.63	0.31
2.16	2.47	0.55	0.36	0.65	0.02	4.28	0.30	2.20	0.97	11.11	0.77	2.63	0.37	76.64	89.92	91.93	−29.82	0.38
35.84	14.57	4.59	7.40	2.05	0.26	32.53	4.94	12.95	8.16	227.64	2.41	20.02	5.57	33.91	28.77	38.59	−30.19	0.38
**Lower Marrat carbonate**																		
74.27	33.31	5.16	0.00	6.88	0.26	70.30	10.24	29.61	9.18	0.00	8.09	43.26	5.53	0.46	0.33	0.46	−29.99	0.43
83.84	18.16	2.66	0.00	10.70	0.13	85.11	11.56	16.14	4.73	0.00	12.59	52.37	2.91	0.46	0.14	0.46	−26.31	0.39
14.83	4.15	1.26	0.00	3.11	0.05	13.22	2.05	3.69	2.24	0.00	3.66	8.13	1.01	1.55	1.55	1.55	−24.15	0.53
0.00	6.11	0.91	0.00	0.00	0.06	17.06	0.00	5.43	1.62	0.00	0.00	10.50	1.29	1.85	1.81	1.85	−25.33	0.44
56.82	11.62	1.91	0.00	3.71	0.14	32.98	7.84	10.33	3.39	0.00	4.37	20.30	2.94	0.61	0.52	0.61	−24.24	0.46
33.59	7.14	1.40	0.00	0.67	0.06	15.86	4.63	6.35	2.49	0.00	0.78	9.76	1.35	2.64	1.82	2.66	−22.39	0.50
20.06	23.39	5.63	0.00	4.50	0.14	41.55	2.77	20.79	10.01	0.00	5.29	25.57	3.11	0.99	0.82	0.99	−23.67	0.76
25.07	14.16	2.27	0.00	1.26	0.11	30.05	3.46	12.58	4.04	0.00	1.48	18.49	2.29	1.22	0.64	1.23	−25.33	0.47
19.36	8.18	2.37	0.00	1.31	0.07	17.98	2.67	7.27	4.21	0.00	1.54	11.07	1.46	1.92	1.89	1.92	−23.83	0.51
15.43	6.36	1.53	0.00	1.19	0.06	14.39	2.13	5.65	2.72	0.00	1.40	8.86	1.22	2.32	1.49	2.34	−25.73	0.51
136.14	53.63	18.32	0.00	99.70	1.06	266.10	18.78	47.67	32.58	0.00	117.29	163.75	22.97	0.06	0.00	0.06	−24.21	0.49

For display purposes, the Al-normalized concentrations are multiplied by 1000. For the raw concentration data, please check the supplementary material.

Calculations of the EFs show that the lower and upper Marrat units have median EF of 10.33 and 8 for Cr, 2.29 and 3.96 for V, and 18.49 and 22.55 for U. In both the lower and upper Marrat units, EF Mo shows no enrichment. Across the middle Marrat, the median EFs of chromium, vanadium, uranium, and molybdenum are 2.91 (range 1.94–18.71), 0.55 (range 0.29–13.53), 4.07 (range 2.23–56.23), and 19.51 (range 6.13–266.70), respectively.

### Alterations and weathering indicators

Several weathering indices, including (i) Chemical Index of Alteration (CIA)^52^; (ii) Chemical Index of Weathering (CIW)^53^; and (iii) Plagioclase index of Alteration (PIA)^54^, were calculated to assess and quantify the degree and extent of weathering across the Marrat Formation (Fig. 2; supplementary material). Significant enrichment of these indicators, up to 2.5 orders of magnitude, was observed in the red beds when compared with the underlying and overlying carbonates. The red beds have a CIA ranging from 2.66 to 79.8 (mean: 59.67), PIA ranging from 0.01 to 95.2 (mean: 66.44), and CIW ranging from 2.73 to 96.5 (mean: 69.75) (Fig. 2). These values are much higher than those of the bounding carbonates. In fact, the carbonates exhibit consistently low values with very similar ranges across these three indicators, ranging from 0 to 3, with a mean value of approximately 1.

### TOC and Paleoproductivity

The TOC content across the Marrat Formation is consistently below 1%, fluctuating between 0.31 and 0.9 wt.%. The average TOC across these sedimentary units is indistinguishable, 0.47 wt.% and 0.51 wt.% between the red beds and carbonate units, respectively (Fig. 2). Paleoproductivity tracers (Ba, Cu, Ni) were also analyzed and their enrichment factors were calculated. In the lower Marrat unit, the median of EF_Ba_, EF_Cu_, and EF_Ni_ are 3.46 (range 0–18.78), 4.04 (range 1.62–32.58), and 3.66 (range 0–117.29), respectively. Similar EF values for the respective elements were calculated for the upper Marrat unit where the medians of these elements were EF_Ba_ 3.40 (range 0–32.23), EF_Cu_ 3.16 (range 0–24.08), and EF_Ni_ 15.20 (range 0–192.32). For the middle Marrat unit, paleoproductivity trace elements exhibit an overall depletion trend compared to the upper and lower units. The median enrichment factors were EF_Ba_ 0.37 (range 0.13–41.17), EF_Cu_ 1.52 (range 0.76–30.78), and EF_Ni_ 1.08 (range 0–2.41).

### Stable organic carbon isotope

The stable δ^13^C_org_ values indicate a pronounced depletion (of up to −5‰) across the middle Marrat red beds relative to the upper and lower Marrat Carbonates. The δ^13^C_org_ values are ranging between −31.17 to −25.16‰ (mean: −29.41‰) for the red beds, while the values across the carbonate units are ranging between −29.99 to −22.53‰ (mean: −24.78‰) (Fig. 2).

## Discussion

### First record of the T-CIE in Arabia

**Figure 3:**
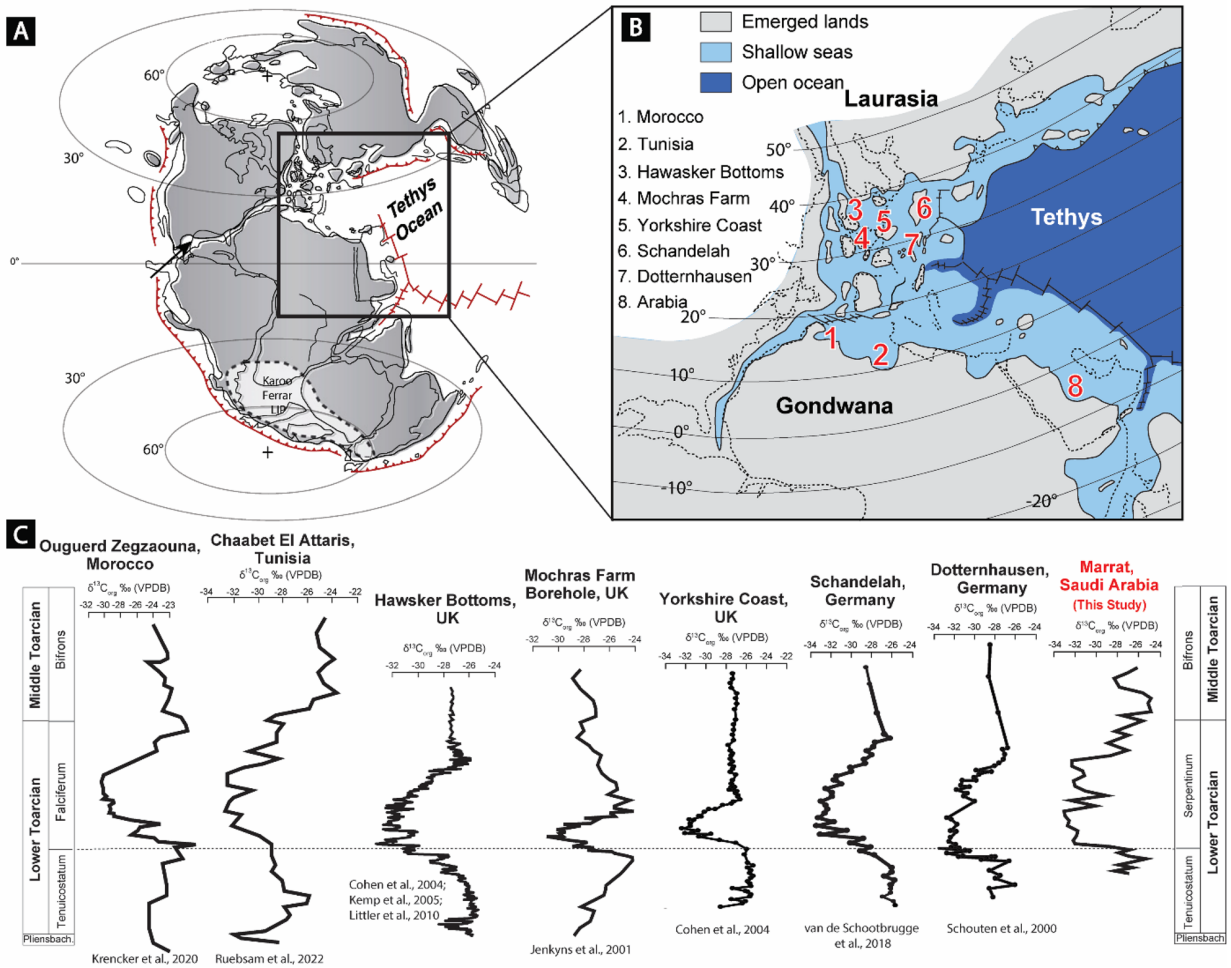
(**A**) Toarcian paleogeographic map of the world^62^. (**B**) Close-up paleogeographic map of the Western Tethyan realm showing the geographic location of Arabia relative to some localities where T-CIE was reported^20^. (**C**) Correlation between the δ^13^C_org_ obtained from the Marrat Formation and the negative excursions in δ^13^C_org_ reported from different basins within the Tethys^6,11,55–60,63,64^.

Several studies have reported the occurrence of negative carbon isotopic excursion (CIE), with a magnitude of −3‰ to −8‰, during the early Toarcian in both the Tethys and Panthalassa oceans (Fig. 3)^2,4–6,11,17–20,55–60^. This Toarcian-aged CIE marked a period of hyperthermal event coupled with the rapid expansion of marine oxygen-deficient areas with severe environmental perturbations^11,61^. Globally, the duration of the T-OAE is constrained between the *tenuicostatum* and *serpentinum* ammonite zones^4,19^. In Arabia, based on ammonite dating, this time interval was constrained to be within the Marrat Formation (Fig. 3)^27–29,45^, suggesting that the Marrat red beds were deposited during the time window of the T-OAE.

However, the recognition of the T-OAE/CIE in Arabia has never been considered due to the traditional non-marine interpretation of these red beds^30–34^, and the lack of δ^13^C_carb_ and δ^13^C_org_ records covering the early Toarcian succession of Arabia. Recently, the newly introduced shallow-marine interpretation for the Marrat red beds raises the necessity to investigate the potential occurrence of the T-OAE/CIE in Arabia^35^.

Our multiproxy approach provides the first systematic geochemical records from the Arabian Toarcian succession. The δ^13^C_org_ records a pronounced CIE, up to 5 ‰VPDB lighter than the bounding carbonates, across the Marrat red beds (Figs. 2 and 3). Based on the ammonite dating of the Marrat carbonates, this negative excursion falls entirely within the expected time window of the T-OAE (Fig. 2). The increase in various weathering indicators (CIA, PIA and CIW) suggests elevated CO_2_ concentrations in the atmosphere and accelerated continental weathering possibly associated with volcanism during the emplacement of Karoo Igneous Province during the early Toarcian^18,65^. Invariance TOC values across this T-CIE zone may be explained by the low preservation potential of organic matter associated with a high-energy depositional environment and increased rate of siliciclastic influx diluting the organic carbon concentration. In fact, and as a result of the low TOC values, major changes in the organo-facies of the Marrat are highly unlikely to cause the negative CIE. Thus, the reported negative CIE within the middle Marrat may indeed reflect the global T-CIE.

### Absence of redox-sensitive trace element enrichment

**Figure 4:**
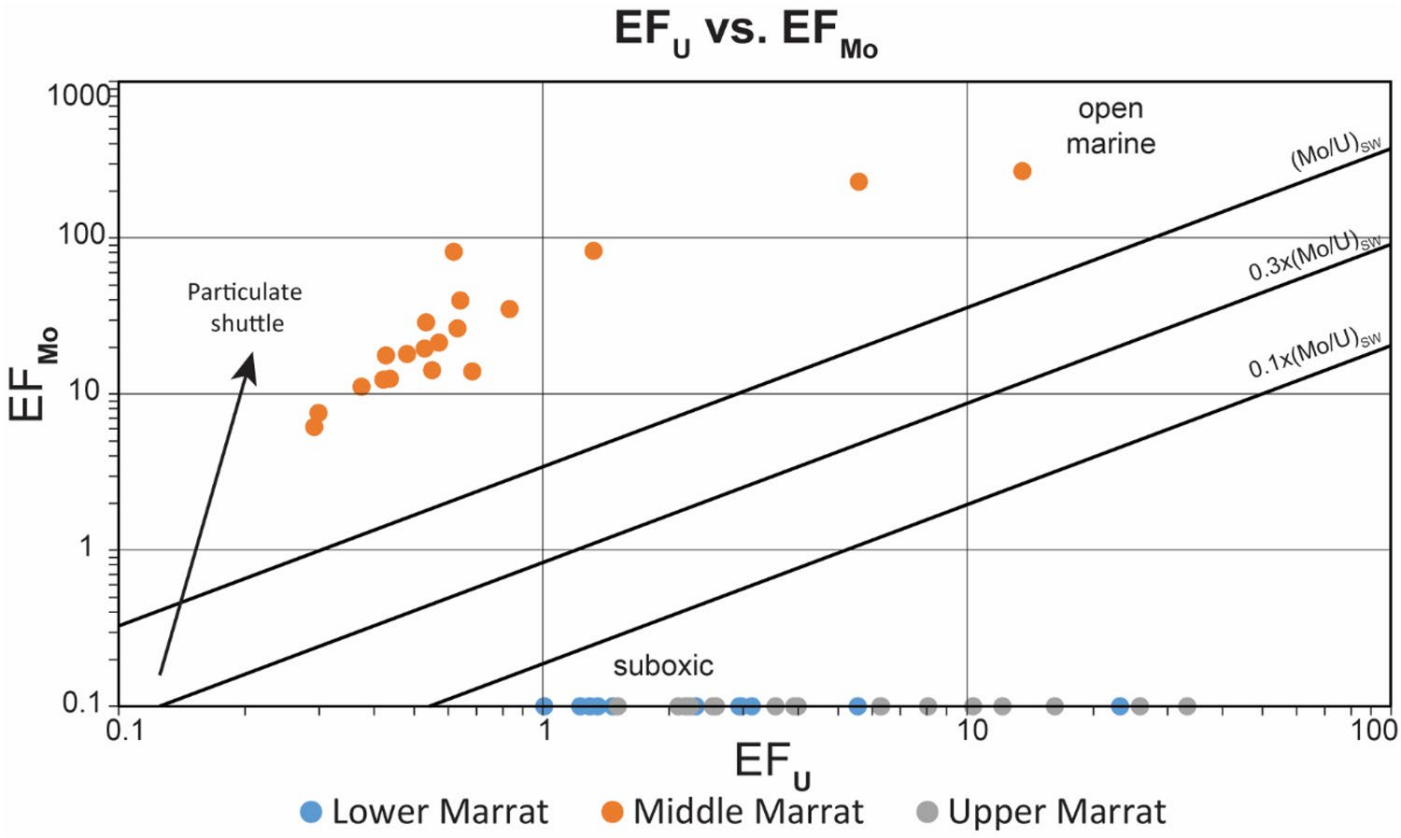
Cross plot of the enrichment factors of Mo and U showing significant enrichment in Mo with no coeval enrichment in U. This trend for the middle Marrat suggests that Mo is likely being scavenged by other phases in the sediment^80,81^ .

Enrichment in redox-sensitive trace elements (such as U, V, Ni, Cu, Mo, Cr) is widely considered as a signal for prevailing redox-conditions during oceanic anoxic events^50,66–74^, however, several studies have highlighted examples for OAE’s where CIE lacks coeval major enrichment in the redox-sensitive trace elements^18,75–78^. Erba et al.^75^ indicate that the mean ocean residence time of many trace elements in the deep oceanic environments can be affected by biological and chemical processes, resulting in controlling the concentrations of these elements in the rock record. On the other hand, for the shallow marine environments with mixed siliciclastic and carbonate sediments, high-energy depositional settings (such as storms) and/or relatively high siliciclastic input are found to be hindering the development of prevailing oxygen-depleted conditions, resulting in the absence of trace-element enrichment^18,78^. The geochemical proxy data of Marrat red beds, except the enrichment factor of Mo, do not exhibit any signature of marine anoxia or increased primary productivity (Fig. 2), suggesting the prevalence of oxic conditions. The enrichment factor of Mo (EF_Mo_) is only anomaly showing significant enrichment of trace elements across the Marrat red beds (Fig. 2). However, the significant enrichment in Mo with no coeval enrichment in U (Fig. 4) may point to particulate shuttle activity^79^. Shuttle of particulate Mo suggests that it is likely being scavenged by other phases in the sediment^80,81^ accelerating the transfer of Mo into the sediment compared to other redox sensitive trace elements.

In general, the observed low concentrations and enrichment factors of redox-sensitive trace elements, along with the low preservation of organic matters, are likely related to the dynamic depositional settings and the elevated siliciclastic input during the middle Marrat time. This possibly had resulted in limiting the upwelling of the oxygen-depleted water to the shallow water settings where the Marrat was deposited along the outcrop. In addition, these findings suggest that marine anoxia is strongly controlled by local to regional basin conditions which make it regional-scale phenomena instead of global phenomena. This is evident from the prevailing development of black shales and marine anoxia in the northern areas of the Tethys shelf, while the southern parts of the shelf were dominated by oxic conditions^3,7^.

It is noteworthy that while Al normalization was performed to minimize or remove the lithological effect on the analyzed elemental data and it yields no enrichment in the red beds, it shows significant enrichment of redox-sensitive metals in the overlying and underlying carbonates (Table1). This suggests a potentially anoxic condition during the deposition of the Marrat carbonates, contrary to the overall environmental conditions and interpretation of these carbonates which have been interpreted to form in a well-oxygenated environment, as indicated by their ^13^C_org_ and fossils abundance. This points to the need of a more robust normalization technique to minimize the lithological effect on the redox-sensitive element concentrations and geochemical proxy (e.g., REE and metal isotopes) to unravel the actual physicochemical ocean conditions during depositions of the Marrat carbonates and red beds.

### Origin and mechanism of reddening

An early interpretation of the Marrat red beds suggested that the reddening or red pigmentation was caused by the enrichment of hematite due to the laterization of the Arabian shield^82^. The majority of previous works agreed that the reddening process of marine red beds was primarily controlled by the presence of iron oxides^83^, with iron being primarily sourced by either continental weathering or biological induction (authigenic precipitation)^22^.

Marine red beds are typically reported as deep, basinal deposits formed under oxic conditions following major OAE’s, particularly during Cretaceous OAE’s^21,22^. Other time periods, including Toarcian, also experienced the development of widespread marine red beds at various time intervals^22,23,84^. Most of these studies interpreted these red beds as basinal deposits that were developed shortly or much later after the T-OAE. In contrast to the deep oceanic red beds, the origin and mechanism of shallow-marine red beds, as in the Marrat Formation, are still much debated. The most widely accepted hypothesis is that shallow-marine red beds developed under oxic conditions, while their counterpart basinal black shale deposits experienced marine anoxia, as illustrated during the Great Ordovician Biodiversification Event^24–26^.

**Figure 5:**
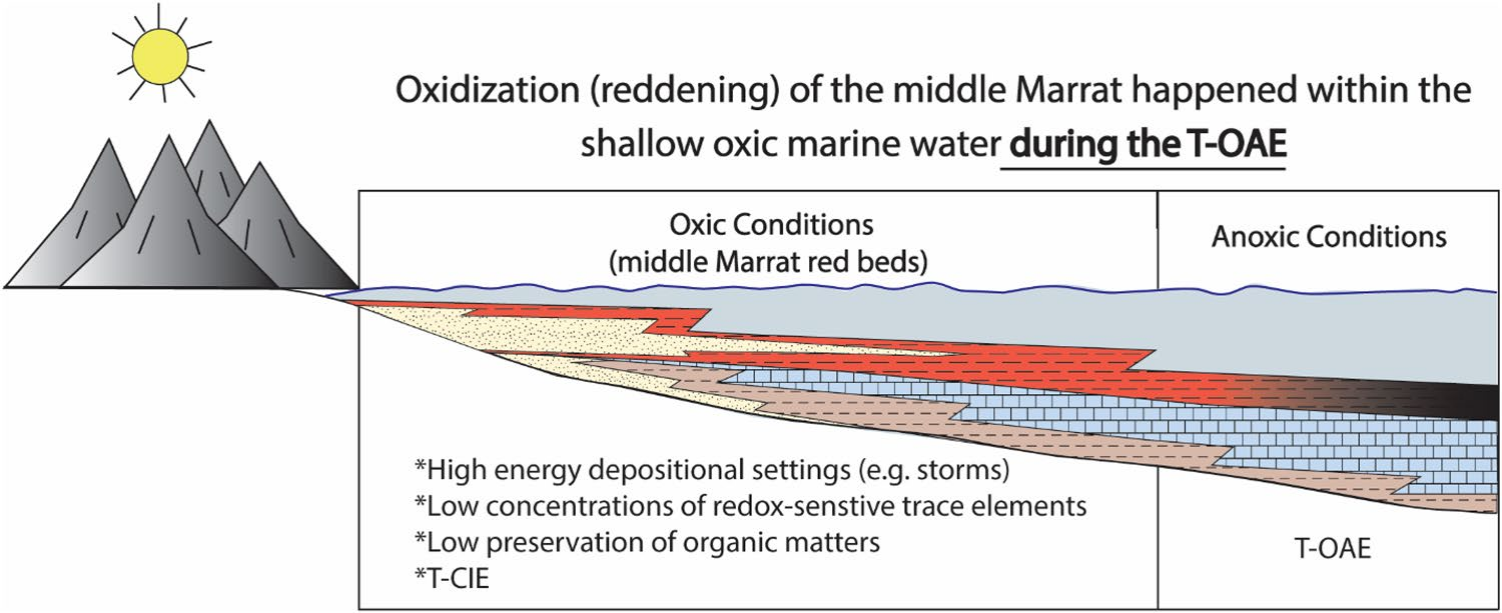
Schematic diagram explaining the proposed model for the middle Marrat red beds, where the redding was possibly taking place in well, oxygenated shallow marine settings ,while the Toarcian oceanic anoxic conditions were dominating the deeper settings.

The Marrat red beds do not exhibit geochemical signals associated with marine anoxia and increased productivity (Fig. 2), proposing shallow marine oxic conditions for the Marrat. It is highly likely due to the intensified continental weathering during the T-CIE, huge amounts of iron were delievered to the Arabian inner shelf, where dominant oxic conditions cauesed their oxidation. In our proposed model, the Marrat red beds were deposited under shallow-water oxic conditions that were time-equivalent to the deep anoxic conditions associated with the T-OAE (Fig. 5).

## Conclusion

The new finding that the middle Marrat deposits are of marine origin provided an opportunity to examine the existence of the Toarcian Carbon Isotope Excursion (T-CIE) in Arabia. Geochemical analyses of the Toarcian Marrat Formation revealed, for the first time, the recognition of the T-CIE in Arabia, which is marked by a distinct negative CIE reported from δ^13^C_org_. Furthermore, the increase in various weathering indicators (CIA, PIA, and CIW) suggests elevated CO_2_ concentrations in the atmosphere and accelerated continental weathering possibly associated with the widespread T-OAE. The observed geochemical signals within the Marrat red beds propose that they were originally deposited in shallow-marine oxic settings, while the Toarcian oceanic anoxic conditions were dominating the deeper settings.

The first recognition of the T-CIE in Arabia, as highlighted in this study, will significantly contribute to the global understanding of this major event and its geographical extent. Furthermore, it will open the door for future researchers to further investigate the occurrence of T-OAE across the Arabian Plate and its potential impact on the Arabian Jurassic stratigraphy which constitutes one of the most prolific petroleum systems in the world.

## Supplementary Information

Supplementary Information.

## Data Availability

All data used in this study is presented here and available in the supplementary material. Additional request may be made directly through the corresponding author on reasonable request.
